# Supplementary figures and images for: Climate-driven models of leptospirosis dynamics in tropical islands from three oceanic basins
Source: PLoS Negl Trop Dis. 2024 Apr 25;18(4):e0011717. doi: 10.1371/journal.pntd.0011717 (PMC11075899; doi:10.1371/journal.pntd.0011717)

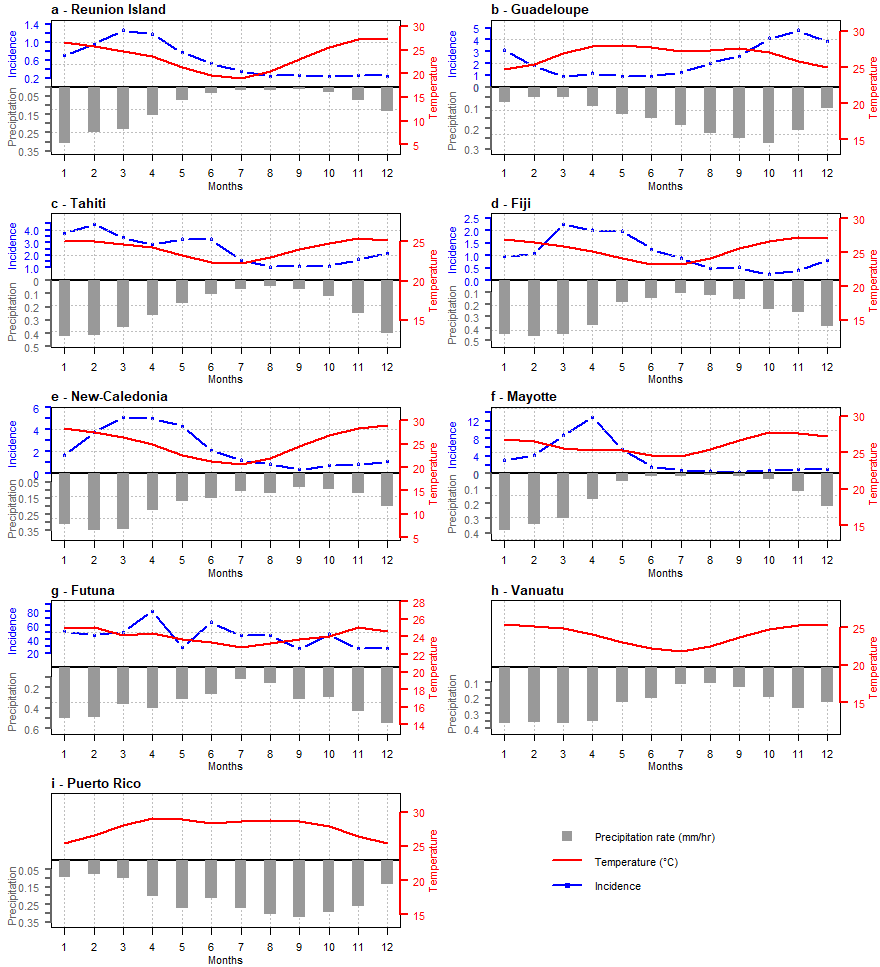

Supplement: S1 Fig — The seasonal profile for Reunion Island (a), Guadeloupe (b), Tahiti (c), Fiji (d), New Caledonia (e), Mayotte (f) and Futuna (g) are defined by the mean temperature (°C—red line) and the mean precipitation rate (mm/hr—grey bar) of each month (left panel). Leptospirosis profile is given in incidence per 100,000 inhabitants and was defined as the median value of incidence of each month (blue line). (TIF) [file pntd.0011717.s001.tif]

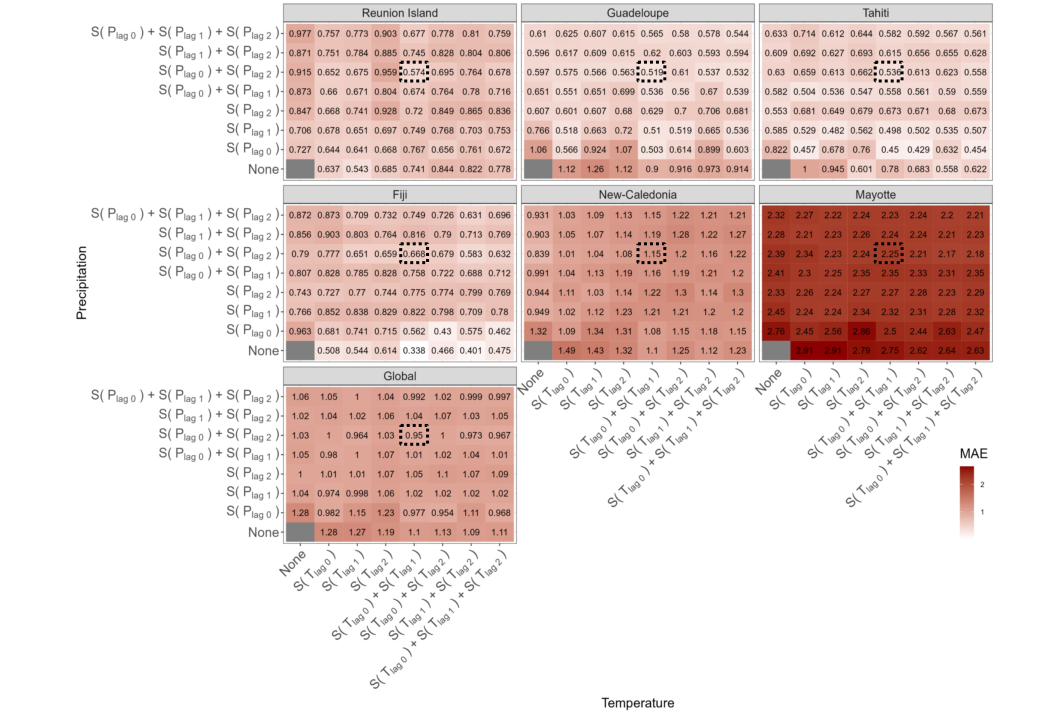

Supplement: S2 Fig — Leptospirosis normalized seasonal profile was estimated based on the normalized seasonal profile of temperature (S(T)) and precipitation (S(P)) with a lag ranging from 0 to 2 months. We performed normalization by removing the mean of the variables in each island. The black dotted boxes frame the best model selected for modelling the leptospirosis seasonal dynamics. (TIF) [file pntd.0011717.s002.tif]

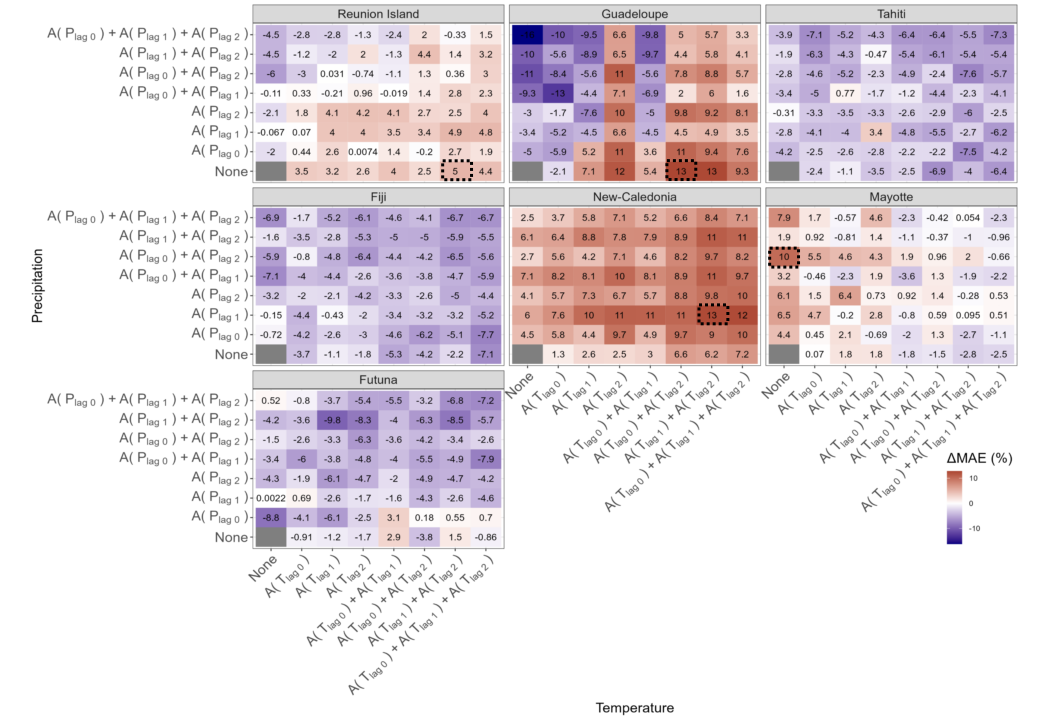

Supplement: S3 Fig — Models estimate the anomalies of log incidence based on precipitation and temperature anomalies (respectively A(P) and A(T)) with a lag ranging from 0 to 2 months. ΔMAE compares the accuracy of the predicted incidence to the seasonal profile. The black dotted boxes frame the best models selected for modelling the leptospirosis anomalies in each island. (TIF) [file pntd.0011717.s003.tif]

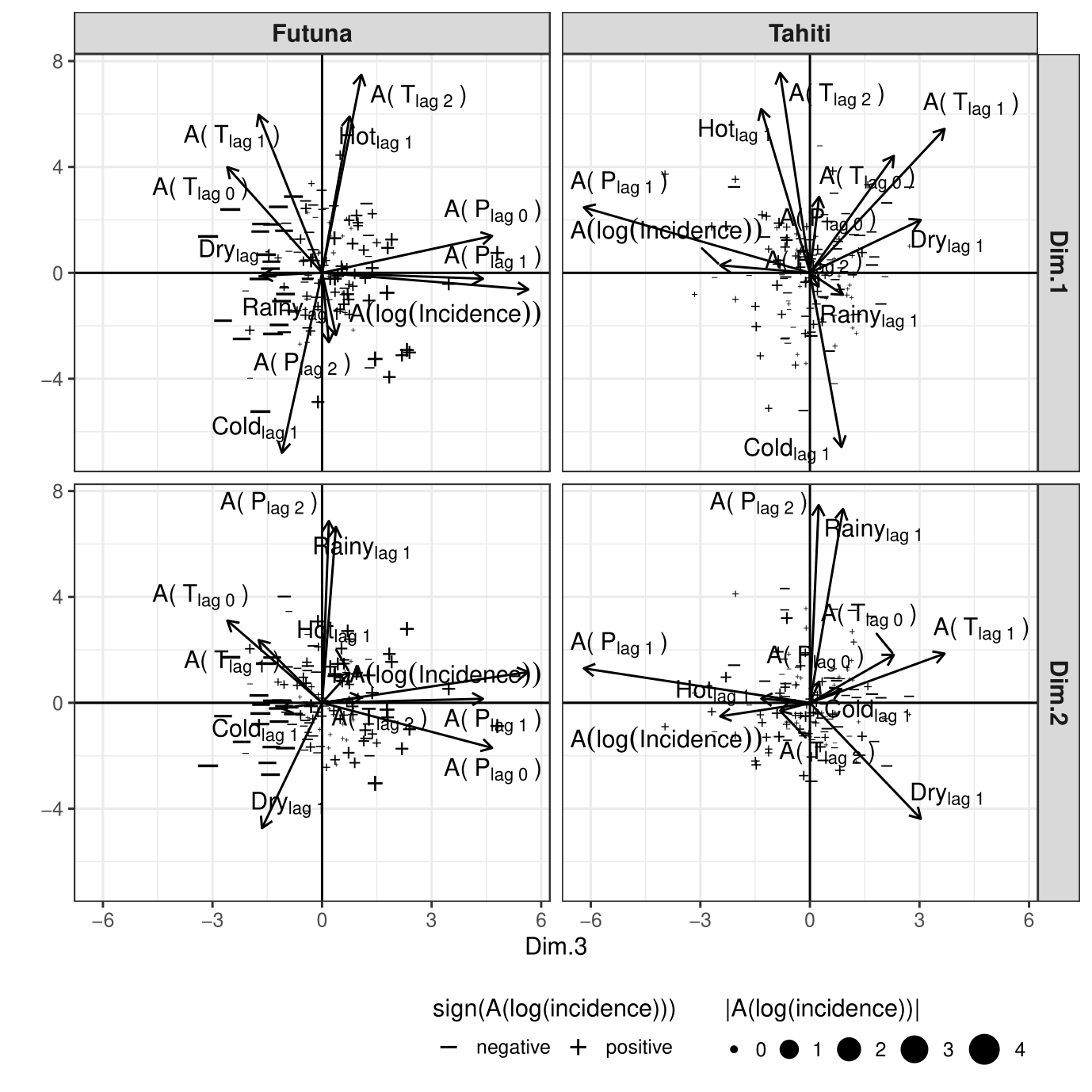

Supplement: S4 Fig — (TIF) [file pntd.0011717.s004.tif]
