# Supplementary material for: Climate-driven models of leptospirosis dynamics in tropical islands from three oceanic basins
Source: PLoS Negl Trop Dis. 2024 Apr 25;18(4):e0011717. doi: 10.1371/journal.pntd.0011717 (PMC11075899; doi:10.1371/journal.pntd.0011717)
Supplement: Attachment — Submitted filename: reviews PLOS NTD.docx [file pntd.0011717.s005.docx]

Response to Reviewers

“**Climate-driven models of leptospirosis dynamics in tropical islands from three oceanic basins”**

**Léa Douchet, Christophe Menkes, Vincent Herbreteau, Joséphine Larrieu, Margot Bador, Cyrille Goarant** ^+^**, and Morgan Mangeas**^+^

^+^Equal senior contribution.

We thank you very much for all your contributions, which have greatly improved our manuscript in both content and form. We have modified the grammar, vocabulary and figure numbering directly in the text according to your suggestions. We completed the conclusion and the figures according to your suggestions. We highlighted all text modifications with a red and underlined writing in the “Ms_LeptoIsland_MarkedChanges” file and addressed the issues raised by your comments below.

# Methods :

## Reviewer #2:

**Table 1. Check the Monthly temperature of Guadeloupe: “3.226.8±1.4”, is it a typing error? Also, subscripts are exchanged. “a” should be monthly precipitation and monthly temperature, whereas “b” should be the population description.**

- It was indeed a typing error; the monthly temperature of Guadeloupe is 26.8 °C. We corrected the values in the table and modified the subscripts (Table 1)

**Line 165: if median is reported and used for time series analysis, why the authors reported only the “mean” in table 1, maybe it is better to report both.**

- We added the information of the median in the table (Table 1).

**Line 173: link has an error. Please replace it.**

- The link has been update and is now working : "<https://gpm.nasa.gov/missions/GPM>” - line 182

# Results

## Reviewer #2:

**Lines 231-232: why if Futuna is one of the islands with the highest incidence it is shown as SM and not in the main manuscript... If the manuscript is about 7 islands, why show the results of only 4?**

- Leptospirosis dynamics was investigated in 7 islands. However, we removed islands that did not match the hypothesis of the models. For example, Futuna was removed from the analysis since the recorded case of leptospirosis on this island does not show a seasonal dynamics rather a highly variable number of cases. For the anomalies model, we presented the islands for which we obtained a good model’s adjustment (in terms of R squared, MAE and ΔMAE) and the islands where we couldn't fit any model were originally presented on the supplementary data. Now ,we updated our manuscript to incorporate all islands on the figures in the main text (Figures 2) and removed it from the supplementary material. We adjusted the text accordingly.

“Although Fiji and Tahiti had a low average incidence (respectively 2±2.9 and 2.7±1.9), both islands observed outbreaks far above the baseline with respective incidence of 20 (year 2013 in Fiji) and 12.6 (year 2010 in Tahiti) (Fig. 2, f and g)” - lines 248-250

“We could not identify climate determinants of anomalies in Tahiti, Futuna and Fiji since all tested models provided poor results in leave-year-out cross validation with low R² scores of respectively 0.21, 0.027 and 0.16 (Fig. 2 and Supplementary Fig. S.3).” - lines 375-377

**Line 233: this should be supplementary material 3. Please reorder according as they appear in the manuscript.**

- We merged the supplementary material 4 with the figure 2 and updated the citations accordingly.

**Line 235: this is statistically different to 2012 or 2014? The difference is 1 per 100,000 approx. as to say "the highest peaks".**

- We re-formulated and detailed the description for leptospirosis dynamics in Guadeloupe:

“ In Guadeloupe (incidence of 2.3±1.8), we observed the lowest incidence between 2014 and 2016, whereas high peaks of incidence recurrently occurred during the rainy season of the other years (Fig. 2, a).” - lines 247-248

**Fig 3. I think it is important to include Futuna in this figure, even if the results are not as expected. Thus, in line 268-269 it is not necessary to refer to the supplementary material, but to the same figure discussed in the paragraph.**

- Recorded incidence of leptospirosis in Futuna was not used in the design of the global seasonal model since we could not observe any seasonal dynamics in this island. We can therefore not include its seasonal dynamics as a leave-island-out prediction. We, however, added the leptospirosis full times serie records of Futuna in the figure 1 and its predicted seasonality (as an independent prediction) in figure 3 with the associated descriptions:

“We then used the model to estimate leptospirosis seasonality in Futuna, Vanuatu and Puerto Rico. For these islands, the predicted seasonal dynamic could be split into two periods: a period of high incidence lasting about 6 months and a period of low incidence for the 6 remaining months (Fig. 3, b). The amplitude of the predicted dynamic was similar for all islands, with an amplitude (maximum incidence - minimum incidence) of 2.3 for Futuna, 2.3 for Vanuatu and of 2.5 for Puerto Rico. In Vanuatu and Futuna, high incidence would occur at the beginning of the year. Leptospirosis incidence in Vanuatu is predicted to reach a peak in May/June. This maximum would be slightly later in the year than in the other islands of the South Pacific Ocean (Tahiti, Fiji and New Caledonia) while it is reached earlier in the year, in February, in Futuna.” - lines 304-312

# Conclusions

## Reviewer #1:

**The conclusions can be improved by pointing out the main findings of the present study and indicating points for future research.**

- The conclusion has been reformulated and completed according to your comments :

“Despite the complexity of the relationship between humans, environment, and reservoir animals, our results emphasize the importance of past and current climate factors, such as, temperature, and precipitation (lagged from 0 to 2 months), in the dynamics of leptospirosis. The key finding of this study is the identification of a unique climate-based model capable of describing the seasonal dynamics of leptospirosis in tropical islands across three oceanic basins on a global scale. This suggests that the seasonality of leptospirosis can be captured solely by climate indicators.

However, at the local scale, accurately estimating leptospirosis burden in tropical islands remains a significant challenge due to the diversity of lifestyles and of environments that influence the ability of climate to trigger outbreaks. While climate factors can easily estimate the seasonality of leptospirosis in tropical islands, a deeper understanding of local specificities, such as human behavior and environment characteristics should be investigated to improve our understanding of the occurrence of outbreaks inter-annually. The identification of climate indicators linked to leptospirosis dynamics represents a crucial initial step toward developing forecasting models. Future research should involve the investigation of a model at finer scale to capture the relationship between climate and disease dynamics at the local level and lay the foundations for the development of early warning systems for leptospirosis epidemics. Such models would inform decision-makers in the health sector and contribute to epidemic preparedness and management. ” - Conclusion

## Reviewer #2:

**Lines 418-423: discuss further the implications of these factors, as was done for social factors in lines 424-429.**

- A great diversity of mammalian species can carry pathogenic leptospires and spread the disease in the environment. The type of environment present on islands involves the presence of different species of carrier animals at local scale. Processes involved in contamination in rural areas likely differ from the ones observed in urban areas. That is now commented in the discussion as :

“Pathogenic leptospires have been detected in many mammalian species including domestic animals, livestock and wild animals (31). In many islands the identification of the most common serotypes suggested that rats are the main reservoir of pathogenic leptospires (31). In Reunion island, the dogs were also identified as a major carrier of the bacteria (10). Futuna’s economy relies on pig farming and taro cultivation and the agricultural practice increases human exposure to leptospires, with both rats and humans spreading the bacteria in the environment (11). In Fiji the presence of rats, mice and mongoose at home did not appear as a significant risk factor while the presence of pigs in the community and cattle in the district significantly increased the odds of being infected (30). Livestock and domestic animals are restricted to specific areas (as farms, crops, backyard and private houses) and have an increased contact with the population as farmers, breeders, veterinary and pets sitters. In contrast, the spread of wild animals such as rodents is more difficult to control and contributes to the spread of bacteria in many different environments, from highly urbanized and populated areas to cultivated fields and very remote areas, potentially exposing a wide range of populations. Indication of the land use could therefore inform on the population exposure. The specificities of Leptospira animal reservoirs across islands is therefore likely to modify the local epidemiology of the disease temporally and spatially (31).“ -lines 448-462

**Lines 435-437: the wording seems to refer to the same study, but two are cited (30 and 31).**

- The wording refer to 2 different studies. We re-formulated the sentence to clearly separate the references :

“Leptospirosis in Fiji was associated with outdoor occupations and poverty (30) whereas in New Caledonia most infections occurred during leisure or subsistence activities as hunting, fishing, bathing or swimming (33) and earlier peaks of incidence, caused by activities carried out during summer holidays, occurred in young age classes (34)” - line 473-476

**I suggest reinforcing the conclusion, it is implied that the model is not useful. recommendations for the use of the model? Who should use it? researchers, decision makers? health personnel? etc.**

- The model set the scene for the selection of climate indicators for the design of early warning systems that should be used by decision-makers to inform and help the management of epidemics. We added this information in the conclusion :

“The identification of climate indicators linked to leptospirosis dynamics represents a crucial initial step toward developing forecasting models. Future research should involve the investigation of a model at finer scale to capture the relationship between climate and disease dynamics at the local level and lay the foundations for the development of early warning systems for leptospirosis epidemics. Such models would inform decision-makers in the health sector and contribute to epidemic preparedness and management.” - lines 495-499

**Lines 450-451: any recommendations on the use of this model? The purpose of generating them is to prevent outbreaks? how should the social, geographic, climatic conditions mentioned be interpreted?**

- Such models aim to characterize and explore the relationship between climate and leptospirosis incidence. This represents a first step toward the design of early warning systems. We added a comment in the conclusion to integrate your suggestions.

Future research should involve the investigation of a model at finer scale to capture the relationship between climate and disease dynamics at the local level and lay the foundations for the development of early warning systems for leptospirosis epidemics. Such models would inform decision-makers in the health sector and contribute to epidemic preparedness and management. ” –lines 521-524

# Editorial and Data Presentation Modifications?

## Reviewer #2:

**Line 76: “from week to months”. From one week, or from weeks. Please edit.**

- The accurate sentence:

“Once released into water and soil, leptospires can survive from one week to months in the environment (4)” - line 77-78

**Lines 89-90 how much is this influence? Please add some figures.**

- The influence of flooding is not well characterized and strongly depends on the study setting, the scale of the study and the territories considered. A review estimated an Odd Ratio of 2.19 for floods while gathering a sample of 14 cohort and case-control studies. We added the reference in the introduction :

“Although the effect of floods is not well characterized and depends on the study setting, the considered territory and the scale of the study, a review encompassing 14 case-control and cohort studies identified floodings as a significant risk factor for leptospirosis with an overall odds ratio of 2.19 (7)“ - line 92-95

**Line 109 poorly documented or notifiable. Please clarify.**

- Leptospirosis is both poorly notified and poorly documented. The lack of awareness and notification in many countries prevents the study and the documentation of the disease.

“n addition, leptospirosis is poorly notified and poorly documented in many tropical countries, and in tropical islands in particular, mainly because of a lack of medical awareness and difficulties associated in diagnosis as the unavailability of laboratory testing and the non-specific symptoms confound with other tropical infections (19).” - line 115-118

**Lines 110-111: add some reference to support this statement.**

- We added the reference : “Goarant, C. (2016). Leptospirosis: risk factors and management challenges in developing countries. Research and reports in tropical medicine, 49-62.” - line 118

**Line 274. Please check and reorder all the supplementary material.**

- There were indeed some inaccurate numbers. We checked all the figures from both the main and supplementary material to reorder the numbering of all figures and tables.
